# The coordinated regulatory impact of AcsS and TpdA on biofilm formation in *Vibrio parahaemolyticus*

**DOI:** 10.3389/fmicb.2025.1652011

**Published:** 2025-08-20

**Authors:** Bin Ni, Jingyang Chang, Yining Zhou, Wanpeng Li, Zhukang Tian, Renfei Lu, Yiquan Zhang

**Affiliations:** ^1^Department of Laboratory Medicine, School of Medicine, Jiangsu University, Zhenjiang, China; ^2^Department of Clinical Laboratory, Children’s Hospital of Soochow University, Suzhou, China; ^3^Department of Clinical Laboratory, Nantong Third People's Hospital, Affiliated Nantong Hospital 3 of Nantong University, Nantong, China; ^4^Health Commission of Qinghai Province, Xining, China

**Keywords:** *Vibrio parahaemolyticus*, biofilm, AcsS, regulation, TpdA, c-di-GMP

## Abstract

*Vibrio parahaemolyticus*, a marine pathogen, employs biofilm formation to enhance environmental persistence and transmission. Biofilm development is intricately regulated by cyclic di-GMP (c-di-GMP), whose levels are controlled by diguanylate cyclases (DGCs) and phosphodiesterases (PDEs). This study elucidates the coordinated regulatory roles of the LysR-type transcriptional regulator AcsS and the PDE TpdA in biofilm formation. Through genetic, transcriptomic, and biochemical analyses, we demonstrate that AcsS promotes biofilm formation by directly activating the exopolysaccharide biosynthesis gene *cpsA* and indirectly repressing *tpdA*, a gene encoding a c-di-GMP-degrading enzyme. Conversely, TpdA inhibits *acsS* expression and antagonizes *cpsA* transcription. RNA-seq revealed that AcsS globally regulates 235 genes, including those linked to flagella, type IV pili, and capsular polysaccharides. Intracellular c-di-GMP quantification showed that AcsS elevates c-di-GMP levels, while TpdA reduces them, establishing a feedback loop. Phenotypic assays confirmed that AcsS-dependent biofilm enhancement operates independently of TpdA, though TpdA partially suppresses biofilm formation in the absence of AcsS. These findings unveil a regulatory circuit where AcsS and TpdA coordinately modulate c-di-GMP metabolism and biofilm-associated gene expression, highlighting them as promising targets for disrupting biofilm-mediated persistence and transmission of *V. parahaemolyticus*.

## Introduction

*Vibrio parahaemolyticus* (*V. parahaemolyticus*) is a bacterium that thrives in marine ecosystems (Baker-Austin et al., 2018). It poses a health risk primarily through the consumption of contaminated seafood, and to a lesser extent, via contact with seawater through small open wounds (Baker-Austin et al., 2018). Additionally, this bacterium is capable of forming biofilms on a range of surfaces, including those found on seafood products (Sharan et al., 2022). Biofilms are bacterial communities encased in a matrix that forms on surfaces and are widely utilized by numerous bacterial species to enhance environmental fitness and facilitate transmission (Yildiz and Visick, 2009; Faruque et al., 2006). The biofilm matrix, which constitutes over 90% of the mass of a biofilm, is predominantly composed of exopolysaccharides (EPS), extracellular proteins, extracellular DNA, and lipids, with EPS being a particularly significant component (Flemming and Wingender, 2010). In *V. parahaemolyticus*, the production of EPS is linked to the *cpsA-K* (VPA1403-1413) and *scvA-O* operons (Liu et al., 2022). Deletion of either operon leads to a decrease in biofilm formation (Liu et al., 2022). Specifically, the *cpsA-K* operon, rather than the *scvA-O* operon, is directly correlated with the transition between wrinkled and smooth colony morphologies in *V. parahaemolyticus*, with the wrinkled variant associated with increased EPS production (Liu et al., 2022). Furthermore, additional structures, including flagella and type IV pili, also influence the biofilm formation of *V. parahaemolyticus* (Yildiz and Visick, 2009).

Biofilm formation is tightly regulated by numerous factors, with the secondary messenger cyclic dimeric GMP (c-di-GMP) being of central relevance. Elevated levels of c-di-GMP typically enhance biofilm formation while simultaneously suppressing motility (Yildiz and Visick, 2009). The synthesis of c-di-GMP is facilitated by the GGDEF domain present in diguanylate cyclases (DGCs), while its degradation is mediated by the EAL or HD-GYP domain found in phosphodiesterases (PDEs) (Jenal et al., 2017). In *V. parahaemolyticus*, proteins with both GGDEF and EAL domains, such as ScrC and ScrG, as well as those with only the EAL domain, like LafV and TpdA, have been shown to serve as PDEs that inhibit biofilm formation and/or promote motility (Boles and McCarter, 2002; Kim and McCarter, 2007; Kimbrough and McCarter, 2021; Martínez-Méndez et al., 2021). Furthermore, proteins harboring the GGDEF domain, including ScrM, ScrJ, ScrL, and GefA, have been identified as DGCs that either promote biofilm formation or inhibit motility (Kimbrough and McCarter, 2021; Kimbrough et al., 2020; Zhong et al., 2022). Additionally, VopY, an EAL domain-containing protein, degrades c-di-GMP, thereby augmenting virulence (Wu et al., 2023). The metabolism of c-di-GMP is intricately regulated by various environmental conditions, including salinity, exposure to antibiotics like chloramphenicol, and the availability of nutrients such as L-arabinose (Li et al., 2021; Zhang et al., 2023; Zhang et al., 2023). Moreover, transcriptional regulators such as OpaR, QsvR, and H-NS modulate c-di-GMP metabolism by regulating the expression of genes encoding DGCs and PDEs (Zhang et al., 2021; Xue et al., 2022; Zhang et al., 2023).

AcsS, a member of LysR-family transcriptional regulators, is significantly regulated by environmental factors, including low-salt growth conditions, the presence of L-arabinose, and incubation time (Zhang et al., 2023; Yang et al., 2010; Zhang et al., 2023). Our recent findings indicate that AcsS enhances the swimming and swarming motility of *V. parahaemolyticus* by activating the expression of genes associated with polar and lateral flagella (Chang et al., 2024), but it inhibits the expression of major virulence determinants, such as thermostable direct hemolysin and type VI secretion system 2, by repressing the transcription of corresponding genes (Ni et al., 2025; Ni et al., 2024). Notably, flagella play a crucial role in the initial stages of biofilm formation and are essential for the development of mature biofilms in *V. parahaemolyticus* (Yildiz and Visick, 2009; Enos-Berlage et al., 2005). Therefore, AcsS is likely involved in regulating biofilm formation in *V. parahaemolyticus*. In this study, our data demonstrate that AcsS coordinates with TpdA to regulate biofilm formation in *V. parahaemolyticus*.

## Materials and methods

### Bacterial strains

The wild type (WT) strain RIMD2210633 of *V. parahaemolyticus* was utilized in this study (Makino et al., 2003). Nonpolar *acsS* deletion mutant (Δ*acsS*), derived from the WT strain, was constructed by our previous study (Chang et al., 2024). The complementation strain Δ*acsS*/pBAD33-*acsS* (C-Δ*acsS*) was constructed by introducing pBAD33-*acsS* into Δ*acsS* (Chang et al., 2024). Control strains (WT/pBAD33 and Δ*acsS*/pBAD33) were generated by introducing pBAD33 into WT and Δ*acsS*, respectively. The *acsS* and *tpdA* double-gene mutant (Δ*acsS*Δ*tpdA*) and the *tpdA* single-gene mutant (Δ*tpdA*) were generated via deletion of a 258-bp fragment (nucleotides 1305–1562) of *tpdA* from Δ*acsS* and WT, respectively, by homologous recombination using suicide plasmid pDS132 (Sun et al., 2012). All primers used in this study are listed in Table 1.

**Table 1:** Primers used in this study.

Target	Primers (forward/reverse, 5′-3′)	References
Construction of mutant		
*acsS*	GTGACTGCAGTTCCACTGACGGTCATCAC/CGATAGGGATAATGCGAAGGGTCTGTTCAAGTGCGATG	Chang et al. (2024)
	CATCGCACTTGAACAGACCCTTCGCATTATCCCTATCG/GTGAGCATGCGTTGTGCCAGCAAGATTTC	
	GTGACTGCAGTTCCACTGACGGTCATCAC/GTGAGCATGCGTTGTGCCAGCAAGATTTC	
*tpdA*	GTGACTGCAGACACCAACACAGGTACATCG/GACGCAGCGTTTCCACTTTCCCTCGTAGAAGAGAAGGGCA	This study
	TGCCCTTCTCTTCTACGAGGGAAAGTGGAAACGCTGCGTC/GTGAGCATGCTGGTAAGCCTGTTCAAACGG	
	GTGACTGCAGACACCAACACAGGTACATCG/GTGAGCATGCTGGTAAGCCTGTTCAAACGG	
Construction of complementation strain		
*acsS*	GATTCTAGAAGGAGGAATTCACCATGGATATCAAACAAC/GTGACTGCAGTTATCGATTAAATATG	Chang et al. (2024)
RT-qPCR		
*cpsA*	GAGAGCGGCAACCTATATCG/CGCCACGCCAACAGTAATG	Zhang et al. (2023)
*tpdA*	AGAATCAACCAACACACGAA/CACAATACTGTTGATGGCGTA	This study
LacZ fusion		
*cpsA*	GCGCGAGCTCCTTCCCTGTAAATAAGTCATCC/GCGCGGATCCAAGCGAACTCCATCTCATAAG	Zhang et al. (2023)
*tpdA*	TCGATAAGCCCGAGTGAAT/TGAGTATGCCATTCTTTCAAC	This study
Two-plasmid LacZ fusion		
*cpsA*	GCGCGAGCTCCTTCCCTGTAAATAAGTCATCC/GCGCGGATCCAAGCGAACTCCATCTCATAAG	Zhang et al. (2023)
*tpdA*	TCGATAAGCCCGAGTGAAT/TGAGTATGCCATTCTTTCAAC	This study
EMSA		
*cpsA*	GCGCGTCGACCTTCCCTGTAAATAAGTCATCC/GCGCGAATTCAAGCGAACTCCATCTCATAAG	Zhang et al. (2023)
*tpdA*	TCGATAAGCCCGAGTGAAT/TGAGTATGCCATTCTTTCAAC	This study
16S rRNA	GACACGGTCCAGACTCCTAC/GGTGCTTCTTCTGTCGCTAAC	Zhang et al. (2023)

### Growth conditions

Unless stated otherwise, the cultivation of *V. parahaemolyticus* was conducted as previously described (Lu et al., 2021). Briefly, *V. parahaemolyticus* was grown in 2.5% (w/v) Bacto heart infusion (HI) broth (BD Biosciences, New Jersey, United States) at 37 °C with shaking at 200 rpm for 12 h. The resultant culture was diluted 50-fold into 5 mL HI broth, and then incubated under the same conditions until it reached to an optical density at 600 nm (OD_600_) value of 1.4. This culture was referred to as the bacterial seed. Subsequently, the bacterial seed was diluted 1,000-fold into 5 mL of HI broth for a third round of growth and was harvested at an OD_600_ value of 1.4. When necessary, the medium was supplemented with 50 μg/mL of gentamicin, 5 μg/mL of chloramphenicol and/or 0.1% (w/v) L-arabinose.

### Crystal violet staining assay

Crystal violet (CV) staining assay was performed similarly as previously described (Zhang et al., 2023). Briefly, the bacterial seed was diluted 50-fold into 2 mL of Difco marine (M) broth 2216 (BD Biosciences, New Jersey, United States), supplemented 5 μg/mL chloramphenicol and 0.1% L-arabinose, in a 24-well cell culture plate, and then incubated at 30 °C with shaking at 150 rpm for 24 h. Planktonic cells were collected for measurement of OD_600_ values. Surface attached biofilm cells were washed with deionized water, and then stained by 0.1% CV. Bound CV was dissolved in 2.5 mL of 20% acetic acid, and then the OD_570_ values were measured. The capacity for biofilm formation was expressed as the ratio of OD_570_ to OD_600_.

### Colony morphology assay

For the colony morphology assay (Zhang et al., 2023), the overnight bacterial culture was diluted 50-fold into 5 mL M broth, and it was then statically incubated at 30 °C for 48 h. After thorough mixing, 2 μL of the culture was spotted onto an HI plate, or an HI plate supplemented with 5 μg/mL chloramphenicol and 0.1% (w/v) L-arabinose, and incubated at 37 °C for 48 h.

### RNA isolation and RNA sequencing

The WT and Δ*acsS* strains were incubated under the same conditions as the CV staining assay, but without the addition of chloramphenicol and L-arabinose. Three technical replicates were conducted for each strain. Bacterial cells were harvested simultaneously from biofilms and planktonic fractions for the preparation of total RNA, which was extracted using TRIzol Reagent (Invitrogen, Massachusetts, United States) (Zhang et al., 2023). One RNA sample was prepared from each technical replicate. RNA concentration and integrity were determined by a Nanodrop 2000 and the agarose gel electrophoresis, respectively. rRNA removal and mRNA enrichment were performed using an Illumina/Ribo-Zero™ rRNA Removal Kit (bacteria) (Illumina, California, United States). All RNA-related manipulations including RNA extraction were performed in GENEWIZ Biotechnology Co. Ltd. (Suzhou, China). cDNA sequencing was performed on an Illumina Hiseq platform (Zhang et al., 2023; Zhang et al., 2022). Gene expression in Δ*acsS* (test group) was compared with that in WT (reference group). DESeq (v1.12.4) was used to identify the differentially expressed genes (DEGs), filtering for *p* ≤ 0.01 and absolute FoldChange ≥ 2. DEGs were further analyzed using the Gene Ontology (GO), Kyoto Encyclopedia of Genes and Genomes (KEGG) pathway, and Cluster of Orthologous Groups of proteins (COG) database (Zhang et al., 2023; Zhang et al., 2022).

### Intracellular c-di-GMP quantification

Intracellular c-di-GMP was quantified as previously described (Zhang et al., 2023). Briefly, bacterial cells were harvested at an OD_600_ value of 1.4, and then they were resuspended in 2 mL ice-cold phosphate buffered saline (PBS). The bacterial suspension was incubated at 100 °C for 5 min, sonicated for 15 min, and then centrifuged at 9,000 g for 5 min. Total protein and c-di-GMP levels in the supernatant were determined using a Pierce BCA Protein Assay kit (ThermoFisher Scientific, Massachusetts, United States) and a c-di-GMP Enzyme-linked Immunosorbent Assay (ELISA) Kit (Mskbio, Hubei, China), respectively. The c-di-GMP level was expressed as pmol/g of protein.

### Real-time quantitative PCR

Bacterial cells were harvested at an OD_600_ value of 1.4. Total RNA was extracted using TRIzol Reagent (Invitrogen, Massachusetts, United States). cDNA was generated from 1 μg of total RNA using a FastKing First Strand cDNA Synthesis Kit (Tiangen Biotech, Beijing, China). Real-time quantitative PCR (RT-qPCR) was performed using a LightCycler 480 (Roche, Basel, Switzerland) together with SYBR Green master mix (Tiangen Biotech, Beijing, China) (Gao et al., 2011). The relative expression levels of each target gene were determined using the 2^−ΔΔCt^ method, with the 16S rRNA serving as the internal control.

### LacZ fusion and β-galactosidase assay

The regulatory DNA region of each target gene was cloned into pHRP309 harboring a promoterless *lacZ* gene and a gentamicin resistance gene (Parales and Harwood, 1993). Each recombinant plasmid was transferred into WT and its corresponding mutants. Transformants were cultured and lysed to measure the β-galactosidase activity of the cellular extracts using a β-Galactosidase Enzyme Assay System (Promega, Wisconsin, USA). Miller Units representing the β-galactosidase activity was calculated as previously described (Zhang et al., 2023). For the two-plasmid *lacZ* reporter assay (Zhang et al., 2023), the recombinant pHRP309 was transferred into *Escherichia coli* 100 λpir (EC100; Epicenter, Wisconsin, USA) bearing pBAD33-*acsS* or pBAD33. The transformants were cultured in Luria-Bertani (LB) broth containing 0.1% L-arabinose and 20 μg/mL chloramphenicol at 37 °C with shaking at 200 rpm. Bacterial cells were harvested at an OD_600_ value of 1.2, and then lysed to measure the β-galactosidase activity in the cell extracts.

### Purification of 6 × His-AcsS and electrophoretic mobility-shift assay

The coding region of *acsS* was cloned into pET28a (Novagen, Darmstadt, Germany). The recombinant pET28a plasmid was transferred into *E. coli* BL21λDE3 to express the His-tagged AcsS protein (His-AcsS). Expression and purification of His-AcsS were performed as previously described for His-OpaR (Sun et al., 2012). The purity of His-AcsS was confirmed by sodium dodecyl sulfate-polyacrylamide gel electrophoresis. The concentration of His-AcsS solution was determined using a Pierce BCA Protein Assay kit. Purified His-AcsS was stored at −60 °C.

For electrophoretic mobility-shift assay (EMSA) (Zhang et al., 2023), the regulatory DNA region of each target gene was amplified by PCR. EMSA was performed in a 10 μL reaction volume containing 0.5 mM EDTA, 1 mM MgCl_2_, 50 mM NaCl, 0.5 mM DTT, 10 mM Tris–HCl (pH 7.5), 0.625 μg/mL salmon sperm DNA, 100 ng target DNA, and a certain amount of His-AcsS. After incubation at room temperature for 20 min, the binding reactions were visualized on a native polyacrylamide gel, which was stained with ethidium bromide and analyzed using a UV transilluminator.

### Replicates and statistical methods

The CV staining, colony morphology assay, c-di-GMP measurement, LacZ fusion assay, RT-qPCR, and two-plasmid *lacZ* fusion assay were performed at least three times, with at least three technical replicates each time. EMSA for each target gene was performed at least two times, independently. The numerical results were expressed as the mean ± standard deviation (SD). To calculate statistical significance, Student’s *t*-tests or two-way ANOVA with Tukey’s *post hoc* corrections were applied, considering a *p* value of less than 0.05 as significant.

## Results

### AcsS promotes biofilm formation by *Vibrio parahaemolyticus*

**Figure 1:**
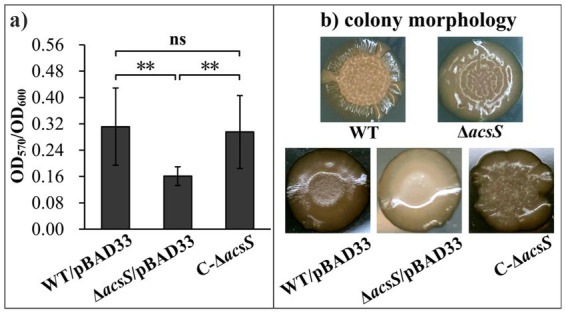
AcsS activates biofilm formation by *V. parahaemolyticus*. Biofilm formation by *V. parahaemolyticus* was assessed using crystal violet staining **(a)** and colony morphology **(b)**. Photographs represent three independent experiments, each with at least three replicates. Two-way ANOVA with Tukey’s *post hoc* corrections were utilized to determine statistical significance. **p < 0.01.

AcsS is involved in promoting the swimming and swarming motility of *V. parahaemolyticus*, which are required for mature biofilm formation (Chang et al., 2024; Enos-Berlage et al., 2005). We therefore investigated the potential regulatory role of AcsS in biofilm formation by *V. parahaemolyticus*. As depicted in Figure 1a, the Δ*acsS*/pBAD33 strain displayed remarkably reduced CV staining compared to both the WT/pBAD33 and C-Δ*acsS* strains (*p* < 0.01). Notably, the C-Δ*acsS* strain demonstrated a restored CV staining pattern indicative of biofilm formation. Additionally, the colony of Δ*acsS* appeared smoother than that of WT (Figure 1b). Moreover, the colonies of both WT/pBAD33 and C-Δ*acsS* appeared smoother than that of WT. This observation may be attributed to the significant influence of chloramphenicol and L-arabinose on this particular phenotype (Zhang et al., 2023; Zhang et al., 2023). While the colony morphology of C-Δ*acsS* and WT/pBAD33 appeared quite different, both exhibited a more wrinkled appearance compared to Δ*acsS*/pBAD33. Since mutation of *acsS* does not affect the growth of *V. parahaemolyticus* (Chang et al., 2024), these results indicate that AcsS positively regulates biofilm formation in *V. parahaemolyticus*.

### Screening for potential target genes of AcsS involved in biofilm formation using RNA-seq

**Figure 2:**
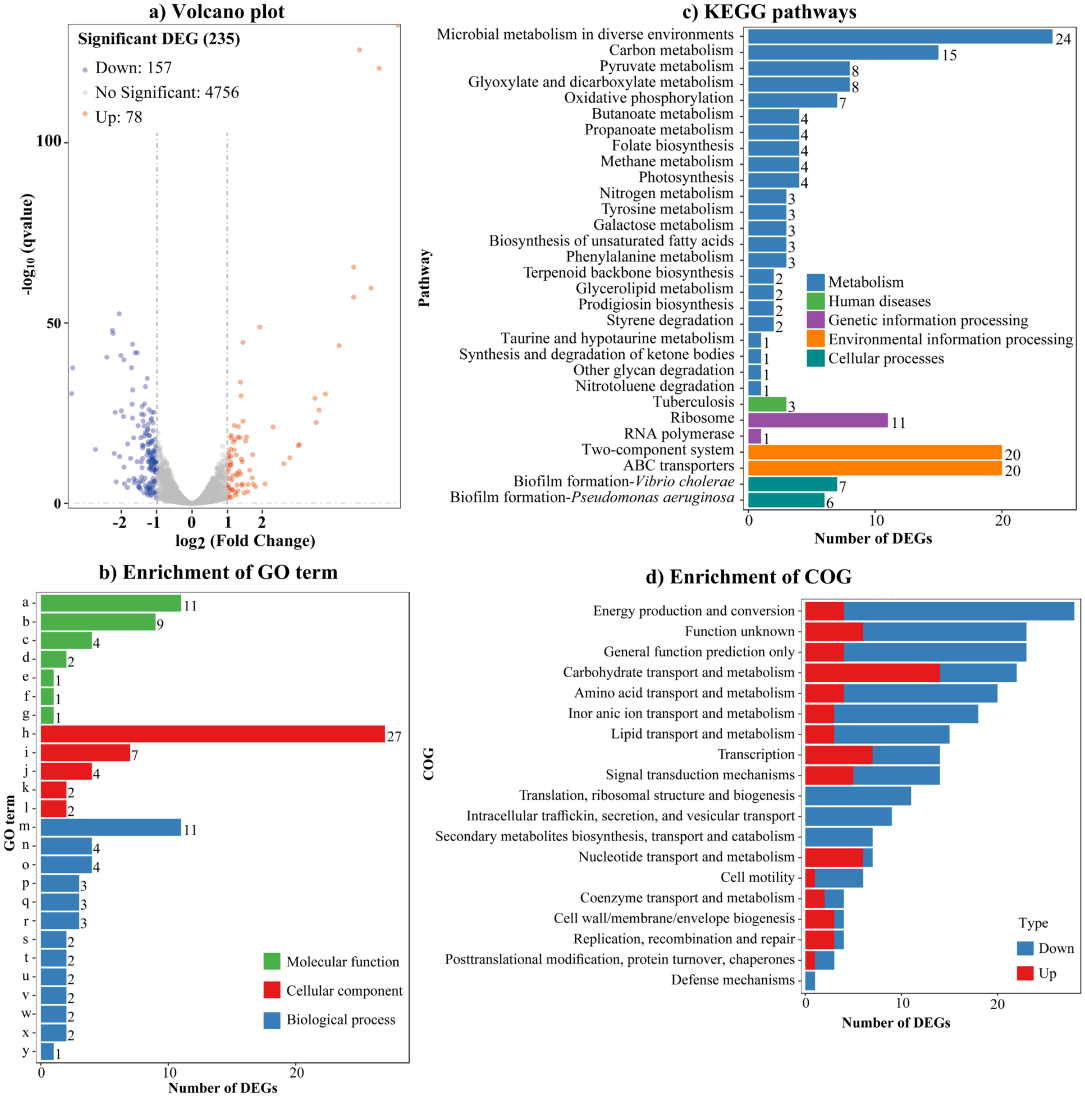
AcsS controls the expression of global genes. **(a)** Volcano plot. Orange, blue and gray points represent the upregulated, downregulated and no-differential expressed genes in Δ*acsS* relative to WT, respectively. **(b)** Enrichment of gene ontology (GO) term. Letters from a-y on the Y axis indicate structural constituent of ribosome, rRNA binding, proton-transporting ATP synthase activity/rotational mechanism, transporter activity, 5-(carboxyamino) imidazole ribonucleotide mutase activity, molybdopterin synthase activity, methylglyoxal synthase activity, plasma membrane, ribosome, proton-transporting ATP synthase complex/catalytic core F (1), small ribosomal subunit, large ribosomal subunit, translation, oxidation–reduction process, “*de novo*” IMP biosynthetic process, carbohydrate transport, ATP synthesis coupled proton transport, phosphate ion transport, valine catabolic process, glyoxylate cycle, lipid catabolic process, L-phenylalanine catabolic process, poly-hydroxybutyrate biosynthetic process, D-gluconate metabolic process, and methylglyoxal biosynthetic process, respectively. **(c)** Enrichment of kyoto encyclopedia of genes and genomes (KEGG). **(d)** Enrichment of cluster of orthologous groups of proteins (COG). The number on the top of each bar in b and c indicates the number of DEGs.

To determine the regulatory mechanism of AcsS on biofilm formation in *V. parahaemolyticus*, RNA sequencing (RNA-seq) analysis was performed comparing the Δ*acsS* (test) and WT (reference) strains. As shown in Figure 2a and detailed in Supplementary Table S1, 235 genes were identified as regulated by AcsS under biofilm growth conditions. Of these, 78 genes were upregulated and 157 genes were downregulated, in Δ*acsS* compared to WT. GO term enrichment analysis indicated that DEGs were associated with molecular functions (7 GO terms, 29 DEGs), cellular components (5 GO terms, 42 DEGs) and biological processes (13 GO terms, 41 DEGs) (Figure 2b). KEGG pathway enrichment results revealed that 181 DEGs mapped to pathways including metabolism, human diseases, genetic information processing, environmental information processing, and cellular processes (Figure 2c). COG enrichment analysis categorized DEGs into 19 functional groups, with the most significant enrichment in function unknown and metabolism-related categories (Figure 2d). These findings suggested that AcsS regulates global gene expression in *V. parahaemolyticus*.

As listed in Table 2, several DEGs implicated in biofilm formation were identified, including *tpdA*, *flgD*, *flgE*, *motY*, *mshG*, *capF*, and VP0226. Specifically, *tpdA* encodes a trigger PDE involved in biofilm formation and c-di-GMP degradation (Martínez-Méndez et al., 2021). The genes *flgD*, *flgE*, and *motY* are associated with the polar flagellar system, while *mshG* belongs to the type IV pili gene cluster. Additionally, *capF* and VP0226 contribute to capsular polysaccharide (CPS) synthesis. However, AcsS is unlikely to promote biofilm formation solely through polar flagella, type IV pili, and CPS, given the extensive gene networks governing these structures in *V. parahaemolyticus* (Makino et al., 2003). The *cpsA-K* gene cluster, directly associated with the wrinkled colony phenotype and regulated by TpdA (Liu et al., 2022), further underscores this complexity. Consequently, *tpdA* and *cpsA* (VPA1403) were selected as focal genes for subsequent experiments.

**Table 2:** Selected DEGs.

Gene ID	Name	Fold change	Product
c-di-GMP metabolism			
VP1881	*tpdA*	5.84	EAL domain protein
Cell motility			
VP0777	*flgD*	0.48	Flagellar basal body rod modification protein
VP0778	*flgE*	0.50	Flagellar hook protein FlgE
VPA1539	*motY*	2.19	Sodium-type flagellar protein MotY
Type IV pili			
VP2700	*mshG*	0.48	MSHA biogenesis protein MshG
Capsule polysaccharide (CPS)			
VP0225	*capF*	0.44	Capsular polysaccharide biosynthesis protein
VP0226		0.45	Rhamnosyl transferase
Regulatory functions			
VP0080		2.00	Sigma-54 interacting response regulator
VP0350	*calR*	3.99	Leucine transcriptional activator
VP0358		2.04	DeoR family transcriptional regulator
VP0569	*phoB*	0.33	DNA-binding response regulator PhoB
VP0570	*phoR*	0.29	Phosphate regulon sensor protein
VP1244		0.49	Response regulator
VP2387		2.02	DeoR family transcriptional regulator
VP2885	*fis*	0.44	DNA-binding protein Fis
VPA0148	*cpxR*	0.43	Transcriptional regulator CpxR
VPA0149	*cpxA*	0.47	Two-component system sensor kinase
VPA0249		0.39	Transcriptional activator
VPA0251		2.11	LysR family transcriptional regulator
VPA0355		0.32	Transcriptional regulator
VPA1472		2.14	MerR family transcriptional regulator

### Regulation of *tpdA* and *cpsA* by AcsS and TpdA

**Figure 3:**
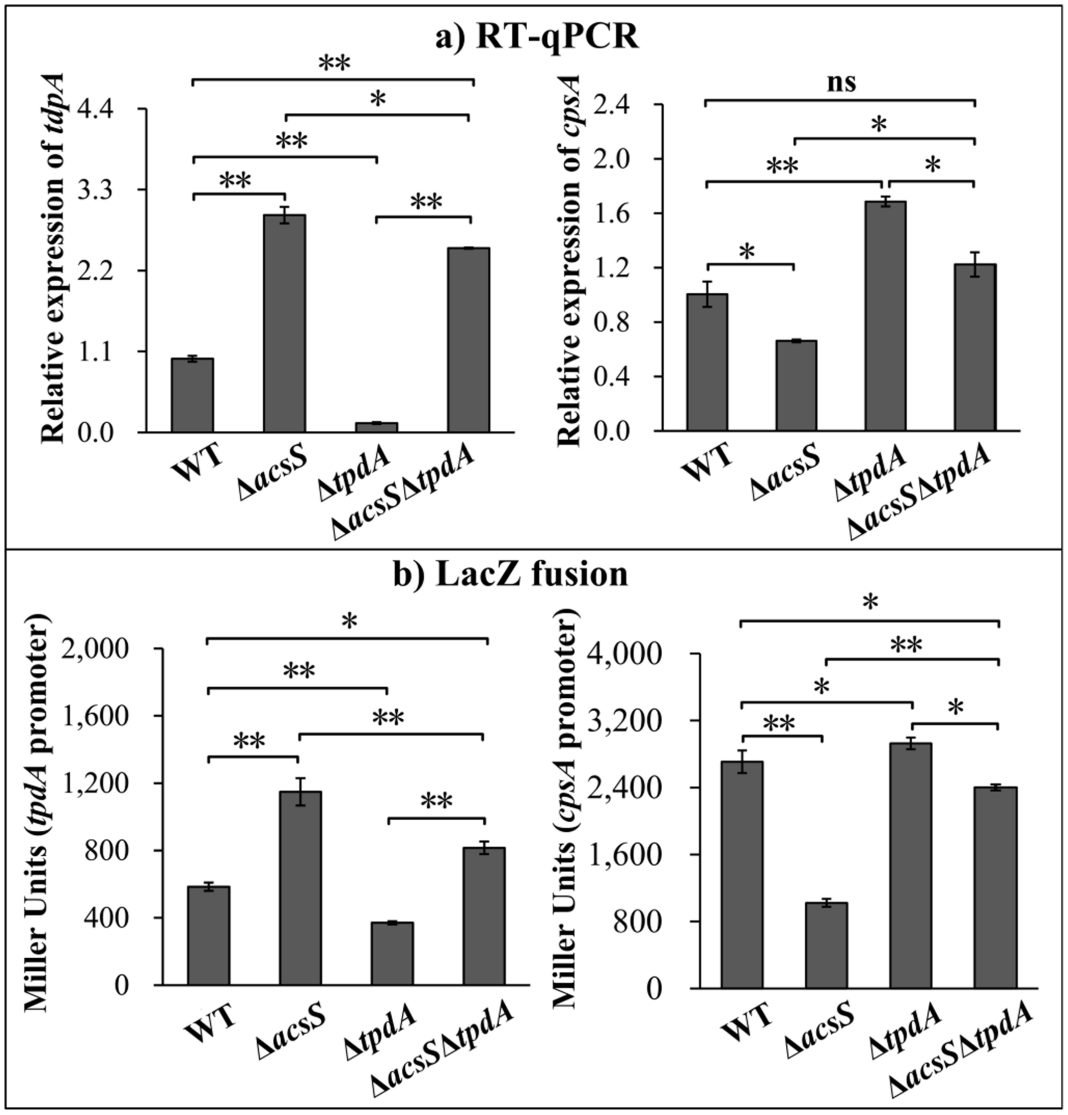
Regulation of *tpdA* and *cpsA* by AcsS and TpdA. *V. parahaemolyticus* strains were grown in HI broth, and bacterial cells were harvested at an OD_600_ value of 1.4. Two-way ANOVA with Tukey’s *post hoc* corrections were utilized to determine statistical significance. *p < 0.05. **p < 0.01. **(a)** RT-qPCR. The relative mRNA levels of each target gene were examined and compared between the WT, Δ*acsS*, Δ*tpdA*, and Δ*acsS*Δ*tpdA* strains. **(b)** LacZ fusion. The regulatory DNA region of each target gene was cloned into pHRP309 and transferred into indicated strains. This was done to determine the β-galactosidase activities (Miller units) in the cellular extracts.

The RT-qPCR results showed that the mRNA level of *tpdA* significantly increased in Δ*acsS* and Δ*acsS*Δ*tpdA* but decreased in Δ*tpdA* compared to WT (Figure 3a). Specifically, *tpdA* mRNA levels were significantly lower in Δ*acsS*Δ*tpdA* than in Δ*acsS* and significantly higher than in Δ*tpdA* (*p* < 0.05) (Figure 3a). Furthermore, the mRNA level of *cpsA* significantly decreased in Δ*acsS* and increased in Δ*tpdA*, whereas no significant change was observed in Δ*acsS*Δ*tpdA* compared to WT (Figure 3a). Compared to Δ*tpdA*, the *cpsA* mRNA level was significantly elevated in Δ*acsS*Δ*tpdA* but significantly reduced relative to Δ*acsS* (*p* < 0.05) (Figure 3a). As further determined by *LacZ* fusion assay (Figure 3b), the promoter activity of *tpdA* significantly increased in Δ*acsS* and Δ*acsS*Δ*tpdA* but significantly decreased in Δ*tpdA* compared to WT (*p* < 0.01). Additionally, the promoter activity of *tpdA* was significantly lower in Δ*acsS*Δ*tpdA* than in Δ*acsS* and higher than in Δ*tpdA* (*p* < 0.01) (Figure 3b). For *cpsA*, promoter activity significantly decreased in Δ*acsS* and Δ*acsS*Δ*tpdA* but increased in Δ*tpdA* compared to WT (*p* < 0.01). Notably, *cpsA* promoter activity in Δ*acsS*Δ*tpdA* was elevated relative to Δ*acsS* but reduced compared to Δ*tpdA* (*p* < 0.01) (Figure 3b). Both assays demonstrated that *tpdA* expression in Δ*acsS*Δ*tpdA* exceeds WT levels, suggesting that AcsS’s negative regulatory effect on *tpdA* outweighs TpdA’s positive regulation. Regarding *cpsA* expression, a discrepancy was observed between the RT-qPCR and LacZ fusion results: RT-qPCR revealed no significant difference in *cpsA* mRNA levels between Δ*acsS*Δ*tpdA* and WT, whereas LacZ assays showed significantly lower *cpsA* promoter activity in Δ*acsS*Δ*tpdA* than in WT. This inconsistency warrants consideration. Possible explanations include: (Baker-Austin et al., 2018) the *lacZ* fusion construct might lack regulatory elements present in the native chromosomal context that modulate mRNA stability or post-transcriptional processing, leading to a discrepancy between promoter activity measured by the reporter and steady-state mRNA levels; or (Sharan et al., 2022) post-transcriptional regulatory mechanism (e.g., affecting mRNA stability or translation efficiency) could differentially influence the endogenous *cpsA* mRNA measured by RT-qPCR versus the heterologous *lacZ* mRNA transcribed from the *cpsA* promoter fusion. Collectively, despite this discrepancy for *cpsA* in the double mutant, the results consistently indicate that AcsS suppresses *tpdA* expression but activates *cpsA*, independent of TpdA. Conversely, TpdA promotes its own expression while repressing *cpsA*, regardless of AcsS.

### AcsS indirectly represses *tpdA* transcription but directly activates *cpsA* transcription

**Figure 4:**
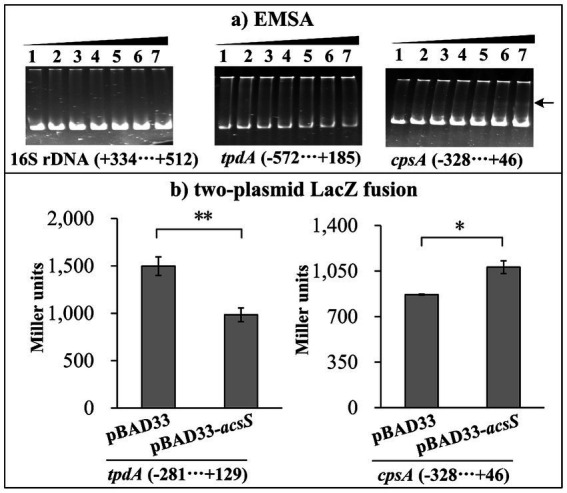
AcsS directly inhibits *tpdA* but indirectly regulates *cpsA*. Negative and positive numbers in brackets indicate the nucleotide positions upstream and downstream of each target gene, respectively. **(a)** EMSA. The regulatory DNA region of each target gene was incubated with increasing amounts of purified His-AcsS and then subjected to 6% (w/v) polyacrylamide gel electrophoresis. DNA bands were visualized using EB staining. Lanes 1 through 7 contain 0.0, 0.8, 1.6, 2.4, 3.2, 4.0, and 4.8 pmol of His-AcsS, respectively. The arrow indicates the shifted band. **(b)** Two-plasmid *lacZ* fusion assay. The plasmid pBAD33-*acsS* (or pBAD33) and a recombinant *lacZ* plasmid were simultaneously introduced into the *E. coli* strain 100 λpir (Epicenter). The promoter activities, measured in Miller units, of each target gene within the cellular extracts were determined using a β-Galactosidase Enzyme Assay System (Promega, United States) according to the manufacturer’s instructions. Student’s *t*-tests were utilized to determine statistical significance. **p < 0.01. ns, *p > 0.05.

The results of EMSA showed that His-AcsS dose-dependently binds to the regulatory DNA fragment of *cpsA* but does not bind to the regulatory DNA region of *tpdA* or the coding region of 16S rRNA (used as a negative control) (Figure 4a). Additionally, a two-plasmid *lacZ* reporter assay demonstrated that expressing *acsS* from pBAD33-*acsS* in EC100 significantly decreased *tpdA* promoter activity while increasing *cpsA* promoter activity (Figure 4b). Although the two-plasmid *lacZ* fusion assay is a widely used method to validate direct regulatory interactions (Zhang et al., 2021; Ante et al., 2015; Bina et al., 2016), results obtained in heterologous hosts like EC100 must be interpreted cautiously. Potential confounding factors include regulator overexpression and incompatibility or unintended interactions between the regulator and the host’s cellular machinery. Collectively, these findings confirm that AcsS directly activates the transcription of *cpsA* and indirectly represses *tpdA* transcription.

### AcsS promotes c-di-GMP production whereas TpdA degrades c-di-GMP in *Vibrio parahaemolyticus*

**Figure 5:**
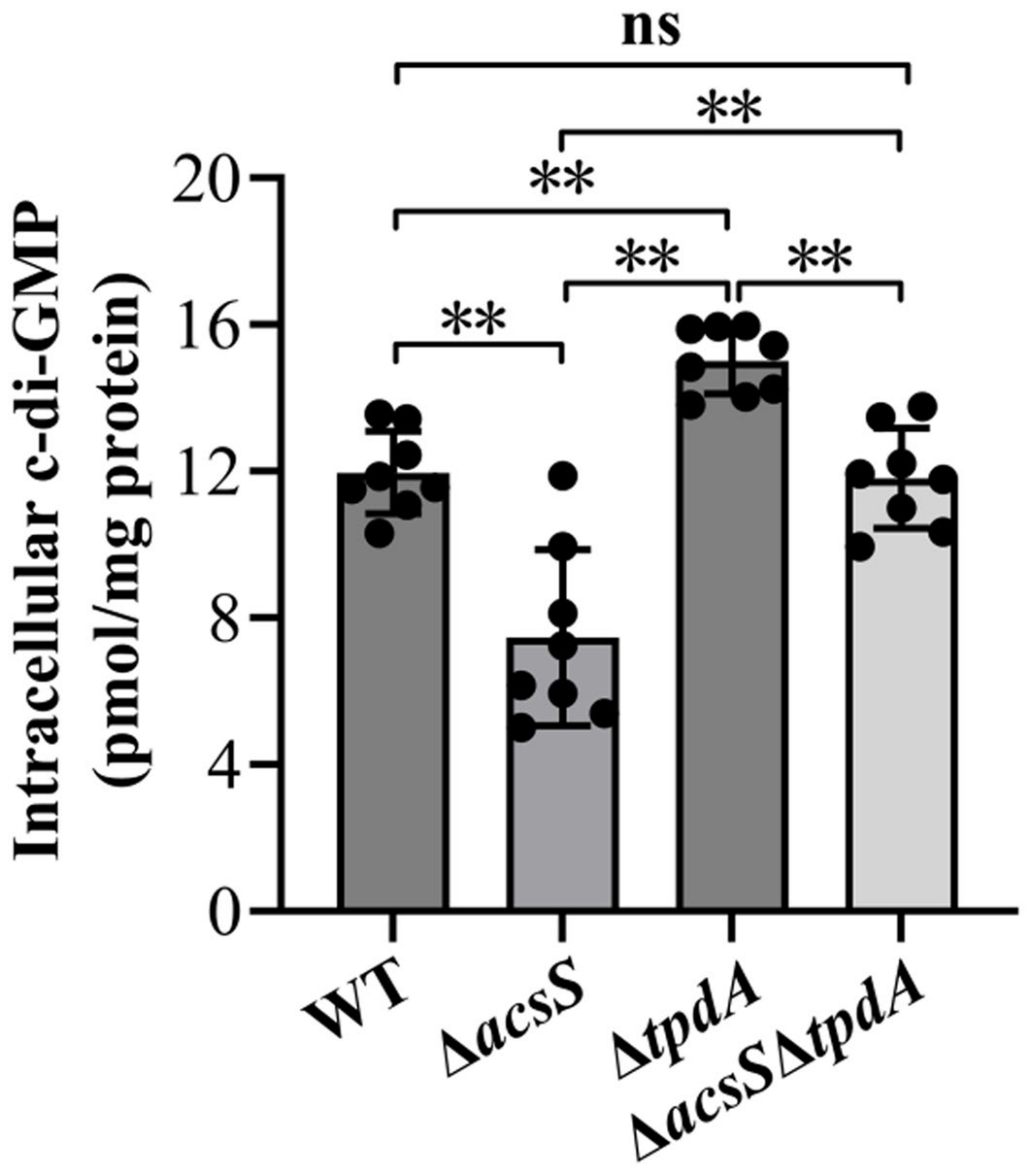
Intracellular c-di-GMP levels in different *V. parahaemolyticus* strains. *V. parahaemolyticus* strains were cultivated in HI broth, and bacterial cells were collected at an OD_600_ value of 1.4. Intracellular c-di-GMP levels were measured using a c-di-GMP enzyme-linked immunosorbent assay (ELISA) kit. The results are presented as the means ± SD from three independent experiments, with each experiment including at least three biological replicates. Two-way ANOVA with Tukey’s *post hoc* corrections were utilized to determine statistical significance. **p < 0.01. ns, *p* > 0.05.

A previous study demonstrated that deletion of *tpdA* led to a 33% increase in c-di-GMP levels compared to WT during exponential growth (Martínez-Méndez et al., 2021). The data from this study also showed that the c-di-GMP level in Δ*tpdA* was significantly higher than in WT (*p* < 0.05) (Figure 5). Additionally, the c-di-GMP levels in Δ*acsS* were significantly reduced compared to WT, Δ*tpdA* and Δ*acsS*Δ*tpdA* (*p* < 0.05) (Figure 3). Furthermore, the c-di-GMP level in Δ*acsS*Δ*tpdA* was significantly lower than that in Δ*tpdA* (*p* < 0.05) (Figure 3). However, no significant differences were detected when comparing Δ*acsS*Δ*tpdA* with WT (*p* > 0.05) (Figure 5). These findings indicate that TpdA degrades c-di-GMP in *V. parahaemolyticus* independently of AcsS, while AcsS stimulates c-di-GMP synthesis regardless of TpdA’s presence.

### AcsS-dependent biofilm formation is independent of TpdA

**Figure 6:**
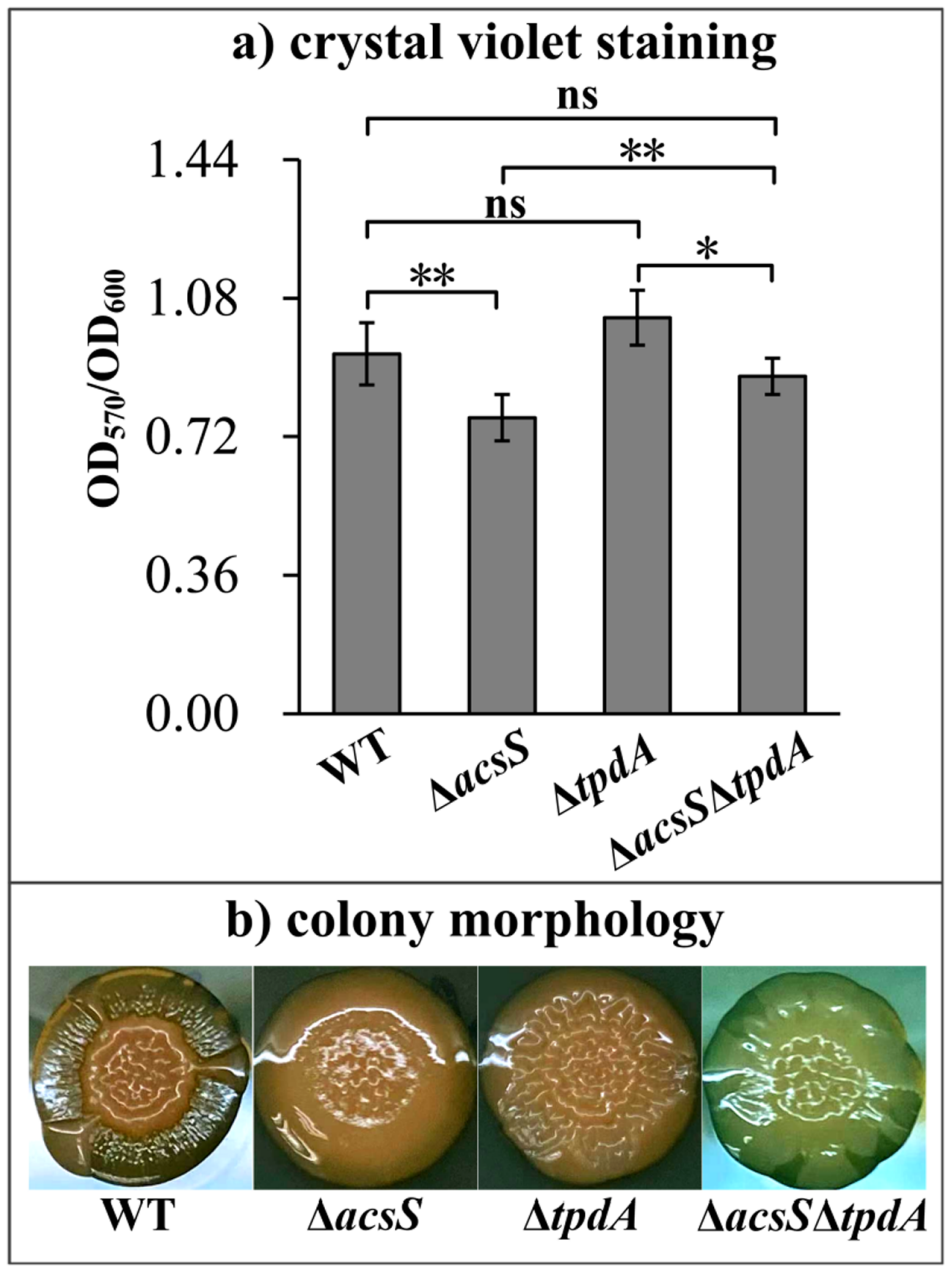
AcsS-dependent biofilm formation was independent of TpdA. The biofilm-forming capacities of the WT, Δ*acsS*, Δ*tpdA*, and Δ*acsS*Δ*tpdA* strains were assessed using crystal violet staining **(a)** and colony morphology **(b)**. Photographs represent three independent experiments, each with at least three replicates. Two-way ANOVA with Tukey’s *post hoc* corrections were utilized to determine statistical significance. *p < 0.05. **p < 0.01. ns, *p* > 0.05.

To determine whether AcsS-dependent biofilm formation is mediated by TpdA, we compared the biofilm-forming abilities of the WT, Δ*acsS*, Δ*tpdA* and Δ*acsS*Δ*tpdA* strains. As depicted in Figure 6a, the Δ*acsS* and Δ*acsS*Δ*tpdA* strains displayed significantly reduced CV staining compared to the WT and Δ*tpdA* strains, respectively (*p* < 0.05). In contrast, the Δ*acsS*Δ*tpdA* strain exhibited significantly enhanced CV staining relative to the Δ*acsS* strain (*p* < 0.01). However, no significant difference was observed between the Δ*tpdA* and WT strains (*p* > 0.05). Additionally, the colonies of WT and Δ*tpdA* were more wrinkled than those of the Δ*acsS* and Δ*acsS*Δ*tpdA* strains (Figure 6b). The colonies of Δ*tpdA* and Δ*acsS*Δ*tpdA* were slightly wrinkled compared to those of the WT and Δ*acsS* strain, respectively (Figure 6b). These results suggest that AcsS-dependent biofilm formation is independent of TpdA, while TpdA appears to partially suppress biofilm formation in the Δ*acsS* genetic background.

### TpdA inhibits the expression of *acsS*

**Figure 7:**
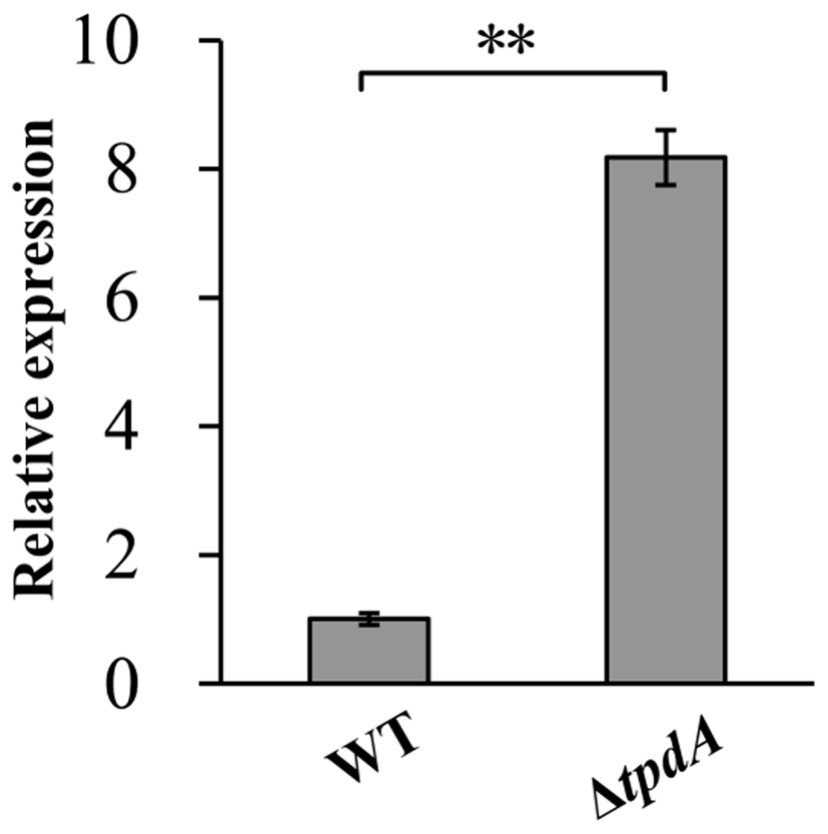
Regulation of *acsS* by TpdA. *V. parahaemolyticus* strains were grown in HI broth, and bacterial cells were harvested at an OD_600_ value of 1.4. The relative mRNA levels of each target gene were examined and compared between the WT and Δ*tpdA* strains. Student’s *t*-tests were utilized to determine statistical significance. **p < 0.01.

To determine whether TpdA regulates *acsS*, we analyzed *acsS* mRNA levels by RT-qPCR. As shown in Figure 7, the mRNA levels of *acsS* were significantly elevated in the Δ*tpdA* strain compared to the WT strain (*p* < 0.01), suggesting that the expression of *acsS* was under the negative control of TpdA in *V. parahaemolyticus*.

## Discussion

LysR-type transcriptional regulators are crucial for a wide range of cellular processes, including metabolism, motility, biofilm formation, and virulence, through their control of gene transcription (Mayo-Pérez et al., 2023). In this study, the data demonstrated that the LysR-type transcriptional regulator AcsS exerts a positive regulatory effect on biofilm formation in *V. parahaemolyticus* (Figure 1). Notably, the expression of AcsS is significantly induced by low-salt growth conditions and L-arabinose, both of which greatly affect biofilm formation in this pathogen (Zhang et al., 2023; Yang et al., 2010). Therefore, further research is necessary to ascertain whether the effects of salinity and L-arabinose on biofilm formation is mediated through the regulation of AcsS.

RNA-seq analysis revealed that AcsS controls 235 genes implicated in a variety of cellular pathways (Supplementary Table S1). However, only a subset of these genes is linked to biofilm formation, including three flagellar genes, one type IV pili-related gene, two CPS biosynthesis genes, and one gene linked to c-di-GMP metabolism (Table 2). Mature biofilm development requires polar and lateral flagella (Yildiz and Visick, 2009; Enos-Berlage et al., 2005). Type IV pili serve as adhesins that facilitate formation of biofilms on surfaces, particularly chitin (Shime-Hattori et al., 2006; Frischkorn et al., 2013). CPS plays a pivotal role in controlling biofilm size by limiting the expansion of mature biofilms (Lee et al., 2013). However, the synthesis of flagella, type IV pili, and CPS involves multiple genes (Makino et al., 2003). It remains unclear whether AcsS has a global impact on the overall synthesis of these structures under the tested conditions, as its regulatory effects appear limited to individual genes within these intricate systems. AcsS indirectly represses the transcription of *tpdA*, which encodes a PDE that degrades c-di-GMP, thereby inhibiting biofilm formation (Martínez-Méndez et al., 2021). In a feedback loop, TpdA inhibits the expression of both *acsS* and its own gene (Figures 3, 4, 7). AcsS-dependent c-di-GMP production may be mediated through TpdA, whereas TpdA inhibits c-di-GMP production independently of AcsS (Figure 5). Moreover, AcsS-dependent biofilm formation is not influenced by TpdA, although TpdA partially inhibits biofilm formation in the Δ*acsS* background (Figure 6). Consequently, AcsS and TpdA coordinately regulate c-di-GMP levels, implicating this signaling molecule as one mechanism through which AcsS controls biofilm formation.

Deletion of *acsS* alone (Δ*acsS*) or in combination with *acsS* and *tpdA* (Δ*acsS*Δ*tpdA*) resulted in smoother colony morphology compared to WT (Figures 1, 6). This phenotype aligns with reduced EPS production (Chen et al., 2010). In *V. parahaemolyticus*, the *cpsA-K* and *scvA-O* gene clusters are responsible for the production of EPS (Liu et al., 2022). However, only the *cps* locus drives EPS phase variation, which mediates transitions between smooth and wrinkled colony morphologies (Liu et al., 2022; Zhang et al., 2022). This variation influences various behaviors of *V. parahaemolyticus*, including motility, biofilm formation, and virulence gene expression (Zhang et al., 2022; Wu et al., 2023). The data presented here showed that AcsS directly activates *cpsA* transcription irrespective of TpdA, while TpdA represses *cpsA* expression independently of AcsS (Figures 3, 4). This antagonistic regulatory relationship suggests that AcsS-mediated control of the *cpsA-K* operon is a key mechanism underpinning its role in biofilm regulation.

Biofilm formation by *V. parahaemolyticus* is intricately controlled by a variety of factors, including nutritional conditions like salinity (Li et al., 2021), metal ion concentrations (Li et al., 2024; Li et al., 2024), and carbon sources (Zhang et al., 2023); environmental parameters like pH (Çam and Brinkmeyer, 2020) and temperature (Billaud et al., 2022); and regulatory proteins such as AphA (Chen et al., 2023), OpaR (Zhang et al., 2021), QsvR (Zhang et al., 2023), OxyR (Chung et al., 2016), CpsQ (Ferreira et al., 2012), ToxR (Chen et al., 2018), and H-NS (Zhang et al., 2018). QsvR directly represses the transcription of *aphA* and *toxR*, while activating *cpsQ* and *opaR* (Lu et al., 2021; Zhang et al., 2019). Furthermore, VPA0607 and *qsvR* are transcribed together as the VPA0607-*qsvR* operon (Zhang et al., 2023). AphA indirectly activates the transcription of VPA0607 at low cell density, whereas OpaR and QsvR directly repress it at high cell density (Zhang et al., 2023). This intricate interplay of regulators is particularly crucial for the precise control of biofilm-related gene expression. In this study, RNA-seq data revealed that AcsS regulates 12 putative regulatory genes, including *calR*, *phoBR*, and *fis* (Table 2). Among these, CalR regulates virulence (Zhang et al., 2017), swarming motility (Gode-Potratz et al., 2010), and biofilm formation (unpublished data). PhoB and PhoR form a two-component signal transduction system (Ortet et al., 2015). In *V. cholerae*, PhoB positively regulates motility and negatively controls biofilm formation and c-di-GMP production (Pratt et al., 2009). In *V. parahaemolyticus*, PhoR is involved in regulating the expression of 1,122 genes, including those responsible for lateral flagella (Zhang et al., 2020). *V. parahaemolyticus* Fis functions as a global regulator, influencing a variety of biological processes such as quorum sensing, the modulation of swimming and swarming motility, and metabolic pathways (Tague et al., 2021). These findings suggest that AcsS may interact with CalR, PhoB/PhoR, Fis, and other regulators to form a coordinated network governing biofilm development. Further studies are needed to dissect these potential interactions and their mechanistic roles.

**Figure 8:**
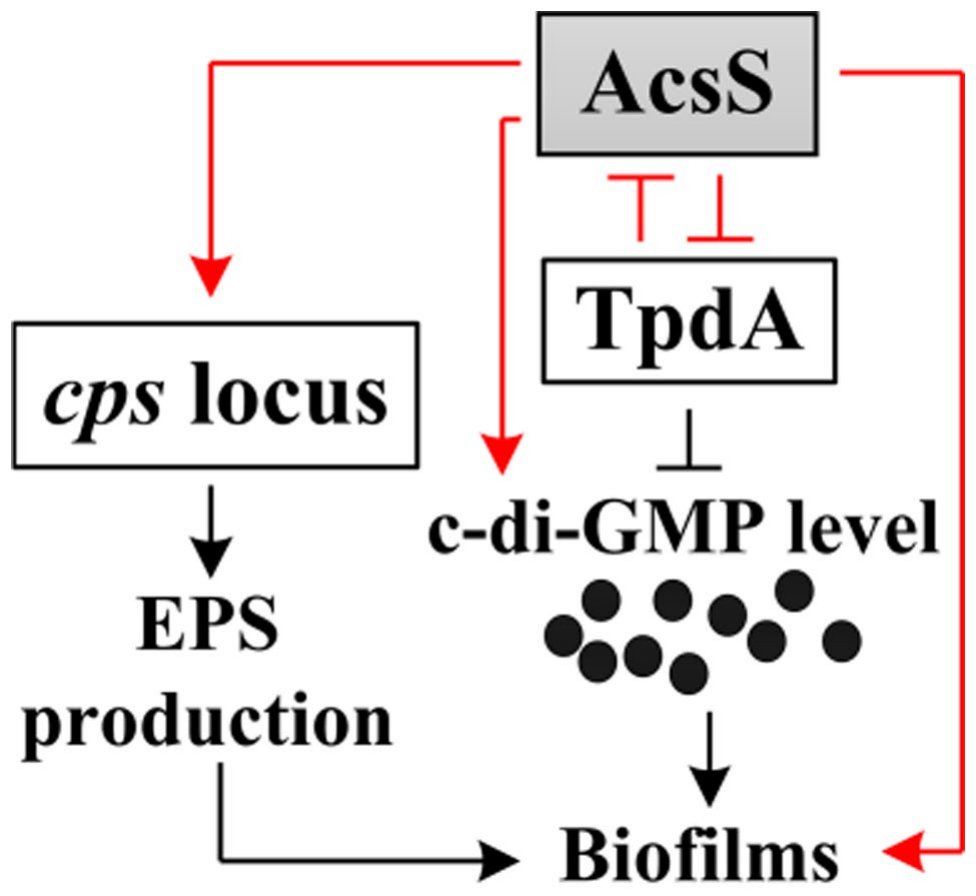
Regulatory circuit. The arrows signify positive regulation, whereas the T-junctions denote negative regulation. The black dots indicate c-di-GMP. The regulatory relationships depicted by red lines are the findings of the current study, while those illustrated by black lines have been established in earlier research (Liu et al., 2022; Martínez-Méndez et al., 2021).

In conclusion, this study demonstrates that AcsS and TpdA coordinately regulate biofilm formation in *V. parahaemolyticus* (Figure 8). AcsS indirectly represses the transcription of *tpdA*, which encodes a PDE that degrades c-di-GMP, thereby promoting the production of c-di-GMP. In a feedback loop, TpdA inhibits the expression of *acsS*. Additionally, AcsS directly activates the transcription of *cpsA* independently of TpdA, while TpdA antagonizes *cpsA* expression. Therefore, AcsS promotes biofilm formation in *V. parahaemolyticus* by regulating the transcription of *cpsA-K* and *tpdA*, as well as the production of c-di-GMP. The data enhance our understanding of the regulatory networks controlling biofilm formation in *V. parahaemolyticus* and highlight AcsS as a key regulator of this process. Importantly, disrupting this regulatory circuit could attenuate biofilm formation, thereby reducing environmental persistence and seafood contamination by this pathogen. Future studies should explore small-molecule inhibitors targeting these regulators to validate their translational potential.

## Data Availability

The original data presented in the study are included in the article/Supplementary material. The raw data of RNA-seq have been deposited in the NCBI repository under accession number PRJNA913656.
