# Supplementary material for: The coordinated regulatory impact of AcsS and TpdA on biofilm formation in Vibrio parahaemolyticus
Source: Front Microbiol. 2025 Aug 20;16:1652011. doi: 10.3389/fmicb.2025.1652011 (PMC12405303; doi:10.3389/fmicb.2025.1652011)
Supplement: Supplementary file 1 [file Table_1.doc]

**Table S1. AcsS regulon (all DEGs)**

| **Locus_tag** | **Gene name** | **Fold change** | **Product** |
| --- | --- | --- | --- |
| VP0016 |  | 0.44 | hypothetical protein |
| VP0026 |  | 0.45 | hypothetical protein |
| VP0062 |  | 2.23 | phosphogluconate dehydratase |
| VP0063 |  | 3.28 | thermoresistant gluconokinase |
| VP0064 |  | 2.87 | gluconate permease |
| VP0080 |  | 2 | sigma-54 interacting response regulator transcription regulator protein |
| VP0085 |  | 0.45 | hypothetical protein |
| VP0086 |  | 0.42 | hypothetical protein |
| VP0110 |  | 2.3 | cytochrome c4 |
| VP0119 | *glnL* | 2.29 | nitrogen regulation protein NR(II) |
| VP0141 |  | 0.48 | general secretion pathway protein L |
| VP0142 |  | 0.45 | general secretion pathway protein M |
| VP0143 |  | 0.38 | general secretion pathway protein N |
| VP0162 |  | 0.43 | hypothetical protein |
| VP0164 |  | 0.43 | TonB system transport protein ExbD2 |
| VP0165 |  | 0.41 | TonB system transport protein ExbB2 |
| VP0166 |  | 0.42 | TolR |
| VP0167 |  | 0.47 | hypothetical protein |
| VP0172 |  | 0.42 | binding protein component of ABC transporter |
| VP0173 |  | 0.45 | oligopeptide ABC transporter ATP-binding protein |
| VP0174 |  | 0.41 | oligopeptide ABC transporter ATP-binding protein |
| VP0178 | *pyrE* | 2.44 | orotate phosphoribosyltransferase |
| VP0225 |  | 0.44 | capsular polysaccharide biosynthesis protein CapF |
| VP0226 |  | 0.45 | rhamnosyl transferase |
| VP0256 | *rpsJ* | 0.48 | 30S ribosomal protein S10 |
| VP0257 | *rplC* | 0.49 | 50S ribosomal protein L3 |
| VP0258 | *rplD* | 0.49 | 50S ribosomal protein L4 |
| VP0259 | *rplW* | 0.48 | 50S ribosomal protein L23 |
| VP0260 | *rplB* | 0.43 | 50S ribosomal protein L2 |
| VP0261 | *rpsS* | 0.43 | 30S ribosomal protein S19 |
| VP0262 | *rplV* | 0.45 | 50S ribosomal protein L22 |
| VP0263 | *rpsC* | 0.38 | 30S ribosomal protein S3 |
| VP0264 | *rplP* | 0.43 | 50S ribosomal protein L16 |
| VP0265 | *rpmC* | 0.41 | 50S ribosomal protein L29 |
| VP0266 | *rpsQ* | 0.42 | 30S ribosomal protein S17 |
| VP0349 |  | 0.45 | hypothetical protein |
| VP0350 | *leuO* | 3.99 | leucine transcriptional activator |
| VP0358 |  | 2.04 | DeoR family transcriptional regulator |
| VP0365 |  | 0.47 | dihydroxyacetone kinase subunit DhaL |
| VP0372 |  | 0.28 | hemolysin |
| VP0483 | *gltD* | 0.33 | glutamate synthase subunit beta |
| VP0526 |  | 0.4 | NptA protein |
| VP0540 |  | 2.73 | carbon starvation protein A |
| VP0569 |  | 0.33 | DNA-binding response regulator PhoB |
| VP0570 | *phoR* | 0.29 | phosphate regulon sensor protein |
| VP0571 |  | 0.25 | phosphate ABC transporter periplasmic phosphate-binding protein |
| VP0572 |  | 0.49 | exopolyphosphatase |
| VP0583 |  | 0.3 | malate synthase |
| VP0584 |  | 0.24 | isocitrate lyase |
| VP0585 |  | 0.46 | acetoin utilization protein AcuB |
| VP0586 |  | 0.35 | hypothetical protein |
| VP0652 |  | 2.69 | hypothetical protein |
| VP0712 |  | 0.37 | hypothetical protein |
| VP0777 | *flgD* | 0.48 | flagellar basal body rod modification protein |
| VP0778 | *flgE* | 0.5 | flagellar hook protein FlgE |
| VP0840 |  | 2.65 | hypothetical protein |
| VP0937 |  | 2.05 | hypothetical protein |
| VP0962 |  | 2.16 | hypothetical protein |
| VP1040 |  | 0.26 | hypothetical protein |
| VP1041 |  | 0.46 | gonadoliberin III-like protein |
| VP1046 |  | 2.05 | hypothetical protein |
| VP1054 |  | 0.49 | cytochrome d ubiquinol oxidase subunit II |
| VP1055 |  | 0.45 | hypothetical protein |
| VP1097 |  | 0.3 | ATP-dependent dsDNA exonuclease SbcC |
| VP1103 |  | 2.59 | alanine dehydrogenase |
| VP1134 |  | 0.39 | hypothetical protein |
| VP1181 |  | 2.61 | lactonizing lipase |
| VP1244 |  | 0.49 | response regulator |
| VP1252 |  | 0.48 | hypothetical protein |
| VP1253 |  | 0.37 | NifS-like protein |
| VP1263 | *hemH* | 2.15 | phosphoribosylaminoimidazole-succinocarboxamide synthase |
| VP1349 |  | 0.46 | 4-hydroxyphenylpyruvate dioxygenase |
| VP1350 |  | 0.46 | oxidoreductase |
| VP1351 |  | 0.47 | hypothetical protein |
| VP1376 |  | 0.34 | chemotaxis protein CheY |
| VP1409 |  | 3.12 | hypothetical protein |
| VP1486 |  | 0.25 | methyl-accepting chemotaxis protein |
| VP1512 |  | 0.43 | hypothetical protein |
| VP1513 |  | 0.42 | formate dehydrogenase large subunit |
| VP1514 |  | 0.36 | formate dehydrogenase%2C iron-sulfur subunit |
| VP1515 |  | 0.36 | formate dehydrogenase%2C cytochrome b556 subunit |
| VP1516 |  | 0.4 | hypothetical protein |
| VP1557 |  | 0.43 | bacteriophage f237 ORF6 |
| VP1563 |  | 2.21 | hypothetical protein |
| VP1644 |  | 0.31 | PrpE protein |
| VP1645 |  | 0.43 | hypothetical protein |
| VP1652 |  | 3.34 | tricarboxylic transport TctB |
| VP1706 |  | 2.39 | hypothetical protein |
| VP1787 |  | 2.06 | transposase |
| VP1881 | *tpdA* | 5.84 | hypothetical protein |
| VP1904 |  | 2.14 | methyl-accepting chemotaxis protein |
| VP1918 |  | 0.39 | hypothetical protein |
| VP1952 |  | 0.42 | hypothetical protein |
| VP1974 |  | 0.42 | 5-methyltetrahydropteroyltriglutamate--homocysteine S-methyltransferase |
| VP2011 |  | 2.93 | tetrathionate reductase subunit B |
| VP2014 |  | 0.45 | tetrathionate reductase subunit A |
| VP2092 |  | 2.4 | molybdenum cofactor biosynthesis protein E |
| VP2095 |  | 2.15 | molybdenum cofactor biosynthesis protein B |
| VP2163 |  | 0.19 | alkaline phosphatase |
| VP2385 |  | 2.32 | glycerol uptake facilitator protein GlpF |
| VP2387 |  | 2.02 | DeoR family transcriptional regulator |
| VP2397 | *galM* | 0.38 | aldose 1-epimerase |
| VP2398 |  | 0.43 | galactokinase |
| VP2404 | *ebgC* | 0.5 | cryptic beta-D-galactosidase subunit beta |
| VP2405 |  | 0.47 | oxidoreductase |
| VP2422 |  | 0.5 | hypothetical protein |
| VP2467 |  | 2.13 | outer membrane protein OmpU |
| VP2514 |  | 2.25 | carbonic anhydrase |
| VP2543 |  | 0.45 | oxaloacetate decarboxylase%2C beta subunit |
| VP2633 |  | 0.47 | hypothetical protein |
| VP2700 |  | 0.48 | MSHA biogenesis protein MshG |
| VP2718 |  | 2.19 | Na+/H+ antiporter |
| VP2842 |  | 0.44 | fumarate reductase subunit C |
| VP2843 |  | 0.45 | fumarate reductase subunit D |
| VP2857 | *cpxP* | 0.42 | periplasmic repressor CpxP |
| VP2885 | *fis* | 0.44 | DNA-binding protein Fis |
| VP2896 | *purH* | 2.48 | bifunctional phosphoribosylaminoimidazolecarboxamide formyltransferase/IMP cyclohydrolase |
| VP2906 |  | 2.2 | hypothetical protein |
| VP2921 |  | 0.49 | DNA-directed RNA polymerase subunit beta' |
| VP3036 |  | 2.42 | 5-(carboxyamino)imidazole ribonucleotide mutase |
| VP3037 |  | 2.57 | 5-(carboxyamino)imidazole ribonucleotide synthase |
| VP3062 |  | 0.48 | threonine dehydratase |
| VP3068 | *atpC* | 0.38 | ATP synthase F0F1 subunit epsilon |
| VP3069 |  | 0.38 | ATP synthase F0F1 subunit beta |
| VP3070 |  | 0.42 | ATP synthase F0F1 subunit gamma |
| VP3071 |  | 0.46 | ATP synthase F0F1 subunit alpha |
| VPA0070 |  | 2.63 | esterase |
| VPA0133 |  | 0.44 | ABC transporter substrate-binding protein |
| VPA0139 |  | 0.49 | PmbA-like protein |
| VPA0148 |  | 0.43 | transcriptional regulator CpxR |
| VPA0149 |  | 0.47 | two-component system sensor kinase |
| VPA0154 |  | 0.49 | TonB system transport protein ExbD2 |
| VPA0179 |  | 0.21 | hypothetical protein |
| VPA0201 |  | 0.41 | malate synthase |
| VPA0238 |  | 0.37 | ABC transporter ATP-binding protein |
| VPA0239 |  | 0.38 | ABC transporter permease |
| VPA0249 |  | 0.39 | transcriptional activator |
| VPA0251 |  | 2.11 | LysR family transcriptional regulator |
| VPA0253 |  | 2.05 | transport protein |
| VPA0312 |  | 2.06 | hypothetical protein |
| VPA0318 |  | 2.34 | outer membrane protein OmpV |
| VPA0350 |  | 0.34 | ABC transporter ATP-binding protein |
| VPA0351 |  | 0.15 | permease |
| VPA0352 |  | 0.22 | permease |
| VPA0353 |  | 0.09 | hypothetical protein |
| VPA0355 |  | 0.32 | transcriptional regulator |
| VPA0382 |  | 0.37 | sorbitol-6-phosphate 2-dehydrogenase |
| VPA0383 |  | 0.35 | hypothetical protein |
| VPA0384 |  | 0.22 | hypothetical protein |
| VPA0452 |  | 0.45 | cytochrome b561 |
| VPA0465 |  | 2.08 | NAD(P)H oxidoreductase |
| VPA0526 |  | 0.34 | OmpU |
| VPA0560 |  | 0.46 | ABC transporter ATP-binding protein |
| VPA0576 | *phhA* | 0.43 | phenylalanine 4-monooxygenase |
| VPA0579 |  | 0.24 | alkaline phosphatase |
| VPA0604 |  | 0.31 | ABC transporter periplasmic substrate-binding protein |
| VPA0626 | *fabG* | 0.48 | 3-ketoacyl-ACP reductase |
| VPA0660 |  | 0.35 | iron(III) ABC transporter ATP-binding protein |
| VPA0673 | *mgsA* | 2.02 | methylglyoxal synthase |
| VPA0697 |  | 2.66 | nonspecific acid phosphatase |
| VPA0707 |  | 2.49 | delta-9 fatty acid desaturase |
| VPA0773 |  | 0.26 | N-ethylmaleimide reductase |
| VPA0774 |  | 0.32 | lactoylglutathione lyase |
| VPA0823 |  | 2.47 | pyruvate kinase |
| VPA0824 |  | 3 | regulatory protein UhpC |
| VPA0859 |  | 0.48 | lipase |
| VPA0865 |  | 2.53 | hypothetical protein |
| VPA0921 | *pntB* | 0.47 | pyridine nucleotide transhydrogenase |
| VPA0959 |  | 0.39 | formate dehydrogenase oxidoreductase protein |
| VPA0962 |  | 3.3 | formate dehydrogenase oxidoreductase protein |
| VPA0968 |  | 0.48 | 3-hydroxy-3-methylglutaryl-CoA reductase |
| VPA0997 |  | 0.26 | lipase-like protein |
| VPA1025 |  | 0.49 | hypothetical protein |
| VPA1031 |  | 0.43 | hypothetical protein |
| VPA1033 |  | 0.48 | hypothetical protein |
| VPA1034 |  | 0.45 | hypothetical protein |
| VPA1035 |  | 0.39 | hypothetical protein |
| VPA1036 |  | 0.31 | hypothetical protein |
| VPA1037 |  | 0.32 | phosphoprotein phosphatase |
| VPA1038 |  | 0.31 | hypothetical protein |
| VPA1039 |  | 0.4 | hypothetical protein |
| VPA1040 |  | 0.45 | hypothetical protein |
| VPA1041 |  | 0.48 | hypothetical protein |
| VPA1050 |  | 3.5 | hypothetical protein |
| VPA1067 |  | 0.41 | serine transporter |
| VPA1117 | *fabG* | 0.37 | 3-ketoacyl-ACP reductase |
| VPA1118 |  | 0.37 | 3-hydroxyisobutyrate dehydrogenase |
| VPA1119 |  | 0.44 | enoyl-CoA hydratase/isomerase |
| VPA1121 |  | 0.45 | acyl-CoA dehydrogenase |
| VPA1131 |  | 2.01 | periplasmic binding protein-like protein |
| VPA1143 |  | 2.38 | molybdenum containing oxidoreductase |
| VPA1147 |  | 0.42 | phenylacetate-CoA ligase |
| VPA1200 |  | 0.44 | periplasmic nitrate reductase%2C cytochrome c-type protein |
| VPA1201 |  | 0.45 | periplasmic nitrate reductase%2C cytochrome c-type protein |
| VPA1204 |  | 0.46 | acetyl-CoA acetyltransferase |
| VPA1205 |  | 0.44 | acetoacetyl-CoA reductase |
| VPA1241 |  | 2.99 | hypothetical protein |
| VPA1242 | *codB* | 2.04 | cytosine permease |
| VPA1350 |  | 0.5 | hypothetical protein |
| VPA1376 |  | 17.22 | hypothetical protein |
| VPA1387 |  | 23.77 | hypothetical protein |
| VPA1388 |  | 44.57 | hypothetical protein |
| VPA1389 |  | 14.04 | hypothetical protein |
| VPA1390 |  | 14.36 | hypothetical protein |
| VPA1391 |  | 4.21 | hypothetical protein |
| VPA1392 |  | 7.23 | hypothetical protein |
| VPA1393 |  | 8.16 | hypothetical protein |
| VPA1394 |  | 10.12 | transposition protein |
| VPA1395 |  | 15.23 | transposase |
| VPA1396 |  | 10.32 | hypothetical protein |
| VPA1397 |  | 31.67 | acyl-CoA thioesterase |
| VPA1398 |  | 2.78 | hypothetical protein |
| VPA1399 | *malG* | 31.62 | maltose transporter permease |
| VPA1400 | *malF* | 52.31 | maltose transporter membrane protein |
| VPA1401 | *malE* | 89.48 | maltose ABC transporter periplasmic protein |
| VPA1402 |  | 33.85 | maltose/maltodextrin transporter ATP-binding protein |
| VPA1436 |  | 0.49 | iron(III) ABC transporter ATP-binding protein |
| VPA1458 |  | 0.21 | phosphate ABC transporter ATP-binding protein |
| VPA1459 |  | 0.22 | phosphate ABC transporter permease |
| VPA1460 |  | 0.26 | phosphate ABC transporter permease |
| VPA1461 |  | 0.48 | phosphate ABC transporter periplasmic phosphate-binding protein |
| VPA1472 |  | 2.14 | MerR family transcriptional regulator |
| VPA1473 |  | 2.74 | hypothetical protein |
| VPA1524 |  | 0.42 | hypothetical protein |
| VPA1528 |  | 0.36 | hypothetical protein |
| VPA1539 |  | 2.19 | sodium-type flagellar protein MotY |
| VPA1570 |  | 2.7 | hypothetical protein |
| VPA1620 |  | 2.15 | maltodextrin phosphorylase |
| VPA1634 | *potE* | 0.47 | putrescine transporter |
| VPA1663 |  | 0.48 | sugar ABC transporter binding protein |
| VPA1671 | *araH* | 0.36 | L-arabinose transporter permease |
| VPA1673 |  | 0.47 | L-arabinose-binding periplasmic protein |
| VPA1674 |  | 0.41 | ribulokinase |
| VPAt05 |  | 0.28 | tRNA-Ser |
| VPAt14 |  | 2.08 | tRNA-Gly |
